# Versican V1 Overexpression Induces a Myofibroblast-Like Phenotype in Cultured Fibroblasts

**DOI:** 10.1371/journal.pone.0133056

**Published:** 2015-07-15

**Authors:** Jon M. Carthy, Anna J. Meredith, Seti Boroomand, Thomas Abraham, Zongshu Luo, Darryl Knight, Bruce M. McManus

**Affiliations:** UBC James Hogg Research Centre, Institute for Heart + Lung Health, Department of Pathology and Laboratory Medicine, University of British Columbia – Providence Health Care, Vancouver, British Columbia, Canada; Helmholtz Zentrum München, GERMANY

## Abstract

**Background:**

Versican, a chondroitin sulphate proteoglycan, is one of the key components of the provisional extracellular matrix expressed after injury. The current study evaluated the hypothesis that a versican-rich matrix alters the phenotype of cultured fibroblasts.

**Methods and Results:**

The full-length cDNA for the V1 isoform of human versican was cloned and the recombinant proteoglycan was expressed in murine fibroblasts. Versican expression induced a marked change in fibroblast phenotype. Functionally, the versican-expressing fibroblasts proliferated faster and displayed enhanced cell adhesion, but migrated slower than control cells. These changes in cell function were associated with greater N-cadherin and integrin β1 expression, along with increased FAK phosphorylation. The versican-expressing fibroblasts also displayed expression of smooth muscle α-actin, a marker of myofibroblast differentiation. Consistent with this observation, the versican fibroblasts displayed increased synthetic activity, as measured by collagen III mRNA expression, as well as a greater capacity to contract a collagen lattice. These changes appear to be mediated, at least in part, by an increase in active TGF-β signaling in the versican expressing fibroblasts, and this was measured by phosphorylation and nuclear accumulation of SMAD2.

**Conclusions:**

Collectively, these data indicate versican expression induces a myofibroblast-like phenotype in cultured fibroblasts.

## Introduction

Versican is a large, multidomain chondroitin sulphate proteoglycan that is found in the extracellular matrix of many tissues in the body. Versican is critical to cardiac development, as a knockout of this gene results in embryonic lethality due to a failure of the heart and blood vessels to develop [1]. Versican is also upregulated after injury where it forms part of the provisional wound repair matrix [2,3], and accumulates pathologically in a number of chronic inflammatory disorders that affect the heart and blood vessels, including atherosclerosis [4], allograft vasculopathy [5], valvular heart disease [6], and in the infarcted heart [7]. Versican is highly expressed in other chronic conditions in which active tissue remodeling is present, such as cancers [8,9,10], lung and airway inflammation [11,12], and kidney disease [13], among others. Despite the importance of versican to development and disease, the function of this proteoglycan and its role in wound healing remain poorly understood.

In a previous report, we demonstrated that versican is synthesized and secreted into the extracellular matrix following injury to valvular interstitial cells [14]. In the current study, we hypothesized that a versican-rich extracellular matrix alters the phenotype of mesenchymal cells, and thereby determines the adaptive or mal-adaptive outcomes during a wound healing response. To test this hypothesis, we cloned the full length cDNA for the V1 isoform of human versican and expressed the recombinant proteoglycan in mouse fibroblasts. Our data suggest versican increases the expression of N-cadherin, integrin β1, and smooth muscle α-actin, and promotes fibroblast contraction of a collagen lattice. These changes appear to be mediated, at least in part, by increased activation of TGF-β signaling in the versican-transfected cells, as measured by the phosphorylation and nuclear localization of SMAD2. Collectively, these data suggest versican promotes a myofibroblast-like phenotype in cultured fibroblasts.

## Materials and Methods

### Cell culture

Mouse embryonic fibroblasts were purchased from Clontech (product number 630914) and cultured in DMEM (high glucose) containing 10% FBS and 100 U/mL penicillin/streptomycin. Cells were maintained in a humidified incubator at 37°C with 5% CO_2_ and used for experiments between passages 6–12. All experiments were repeated a minimum of 3 independent times.

### Cloning and expression of the Versican V1 isoform

The full length human versican cDNA for the V1 isoform (accession number X15998.1) was synthesized by Epoch Biosciences (Houston, USA) with unique restriction sites for MLUI and NOTI created at the N- and C-termini, respectively. This cDNA was then subcloned into the pTRE2hyg-6xHN vector (Clontech, product number 631053) using the abovementioned restriction sites to generate the versican expression construct. Subcloning was confirmed by digesting this construct with EcoRV, and the full length versican V1 cDNA was sequenced for accuracy. The versican construct (or the empty vector control) was then stably transfected into the mouse embryonic fibroblasts (Clonetech 630914) using Fugene 6 (Roche) according to the manufacturer’s instructions, and clones were selected by adding 500 μg/mL hygromycin to the growth media. Western blot with a monoclonal antibody against the V0/V1 isoforms of human versican (US Biological, product number L1350) was used to confirm expression of the recombinant protein, and clones with high expression of versican were selected for experimental purposes. Stable cell lines were maintained in growth media supplemented with 100 μg/mL G418 and 100 μg/mL hygromycin, which was removed from the growth media for experiments.

### Western blotting

Cell lysates were collected in lysis buffer (10mM HEPES (pH 7.4), 50 mM Na_4_P_2_O_7_, 50 mM NaF, 50 mM NaCl, 5 mM EDTA, 5 mM EGTA, 2 mM Na_3_VO_4_, and 1 mM phenylmethylsulfonyl fluoride, with 0.1% Triton X-100 and 10 μg/mL leupeptin) followed by centrifugation at high speed (14000 X g at 4°C for 10 minutes) to recover proteins. The protein concentration of samples was measured by a Bradford protein assay. Equal amounts of protein from each sample were separated with SDS-PAGE (10% polyacrylamide) and transferred to a nitrocellulose membrane. Membranes were blocked for 1 hour in 5% milk/TBST and incubated overnight at 4°C with primary antibody in 2.5% milk/TBST. Following 3 washes in TBST, secondary antibody (Santa Cruz biotechnology) at a concentration of 1:2000 in 2.5% milk/TBST was added for 1 hour at room temperature. Antibody binding was visualized with the enhanced chemiluminescence detection system (Thermo Fischer Scientific). Images were captured with a Chemigenius2 system (Syngene, Frederick, USA) and band intensities were calculated with ImageJ software. All immunoblots were done in triplicate and repeated a minimum of 3 times. Antibodies used were as follows: N-cadherin (Abcam, product number 18203), integrin β1 (Cell Signaling Technologies, product number 4706), pFAK397 (BD Biosciences, product number 611723), smooth muscle α-actin (Santa Cruz Biotech, product number sc-32251), and pSMAD2 (Cell Signaling Technologies, product number 3108).

For detecting versican by Western blot, the procedure was modified slightly by using non-reducing sample buffer and a 5% polyacrylamide separating gel, followed by incubating the membrane with anti-versican at a concentration of 1:1000 as described above.

### Immunofluorescent staining and confocal microscopy

Cells were fixed for 20 minutes in 3.7% formaldehyde, washed with PBS, permeabilized with 0.1% triton X-100 for 20 minutes, blocked for 30 minutes with 1% BSA in PBS and incubated overnight at 4°C with the indicated primary antibody at a concentration of 1:100 in 1% BSA. Following incubation with primary antibody, cells were washed with PBS and incubated with anti-mouse Alexa-fluor488 or –fluor594 conjugated secondary antibody (Invitrogen) at a concentration of 1:200 in 1% BSA for 1 hour at room temperature in the dark. In certain experiments, cells were washed and then stained for 20 minutes with Alexa-fluor594 phalloidin (Invitrogen) to visualize f-actin. Cells were washed a final time in PBS and coverslipped with VectaSheild mounting medium containing DAPI (Vector Labs). Images were captured using a Leica AOBS SP2 confocal microscope as our laboratory has previously described [15].

### Cell proliferation, migration and adhesion assays

Cell proliferation was measured by MTS assay (Promega) according to the manufacturer’s instructions. Migration was measured using the in vitro scratch wound assay, as previously described [16]. Briefly, cells were grown to confluence and monolayers were scratched using a dental device to create a cell-free area where migration could be measured. Adhesion was measured by trypsinizing and seeding cells into culture dishes at a concentration of 2X10^5^ cells/mL. Cells were allowed to adhere for 30 minutes prior to taking photographs. For migration and adhesion images, cells were photographed with a Nikon 50i series upright microscope equipped with a digital camera.

### Collagen gel contraction assay

Collagen gel contraction was performed as we have previously described [17,18]. Briefly, twelve-well culture dishes were coated with 1% BSA and incubated for 1 hour at 37°C to create a non-stick surface that prevents gels from attaching to the dishes. Cells were trypsinized, counted and seeded into a 0.5 mg/mL type I collagen solution (BD Biosciences, product number 354236) in growth media at a concentration of 1X10^5^ cells/mL. The collagen/cell suspension was then vortexed, and 1 mL per well was added to the BSA-coated dishes and the solution was allowed to polymerize for 45 minutes at 37°C. Fresh growth media was then added to the solidified collagen gels and plates were returned to the incubator. Collagen gel contraction was monitored over a period of 7 days and the surface area of contracted gels was measured using Image-Pro Plus software (Media Cybernetics, Bethesda, USA). The collagen gels were fixed and immunostained as described above.

### qRT-PCR analysis

RNA extraction, cDNA synthesis and qRT-PCR analysis were performed as our lab has previously described [18]. Predesigned primers to smooth muscle α-actin and β-actin were purchased from Applied Biosystems (assay numbers and Mm00725412_s1 and Mm00607939_s1, respectively).

### Statistical analysis

Results are represented as the mean ± standard deviation. Significant differences in treatment groups were determined using the unpaired Student’s *t*-test. For all analyses, *p<0*.*05* was considered statistically significant.

## Results

### Expression and secretion of human versican in mouse fibroblasts

**Fig 1:**
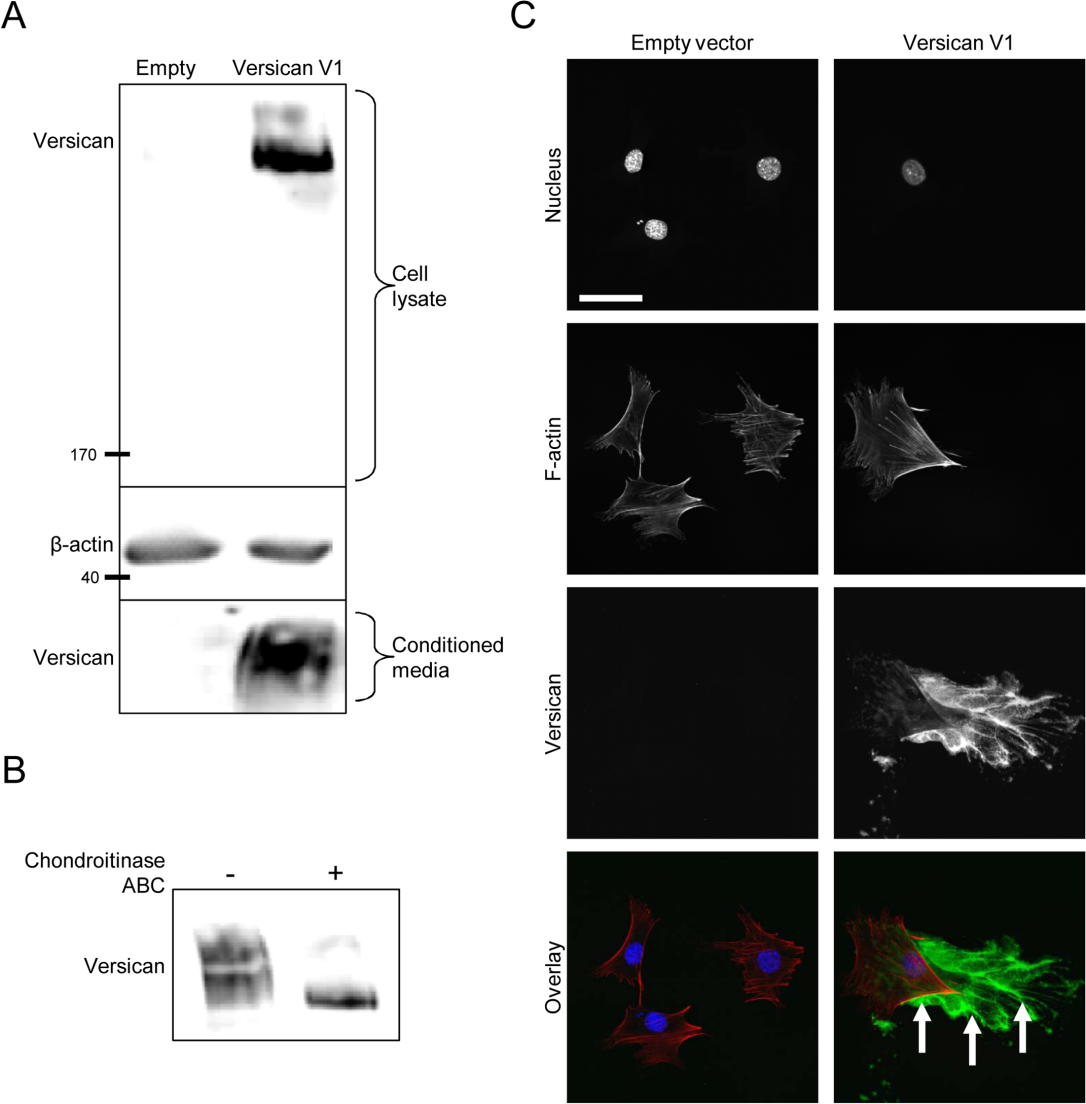
Versican expression in murine fibroblasts. (A) Western blot of recombinant versican expression in the cell lysate and conditioned medium of versican-transfected fibroblasts, suggesting the recombinant protein is synthesized and secreted. (B) Cell lysates were digested with chondroitinase ABC prior to Western blotting to confirm the presence of GAG chains on the recombinant versican. In the absence of chondroitinase, versican appeared as a large smear representing molecules of different molecular weights; after chondroitinase treatment, versican appeared as a compact band at the size of the smallest versican molecules from the smear, suggesting the GAG chains were present and had been removed. (C) Immunofluorescence microscopy showed recombinant versican was deposited into the ECM in versican-transfected cells (green, arrows). (Scale bar = 47.00 μm).

Fibroblasts that had been stably transfected with the human versican V1 construct (or empty vector control) were seeded into 6-well dishes and grown for 48 hours prior to harvesting cell lysates and conditioned media for Western blot. Recombinant versican was detected in the cell lysate and conditioned media of versican-transfected cells, suggesting the recombinant protein is both synthesized and secreted (Fig 1A). As versican achieves much of its function through the GAG chains attached to the central domains of its core protein [19], we next determined if the recombinant versican was synthesized with GAG chains by treating cell lysates with chondroitinase ABC prior to Western blotting. In the absence of chondroitinase treatment, versican appeared as a large smear, representing molecules of different molecular weights with GAG chains attached. After chondroitinase treatment, versican appeared as a tight and compact band running at the size of the smallest versican molecules from the smear, confirming the presence of GAG chains on the recombinant protein (Fig 1B). Immunofluorescent staining showed versican is deposited into the ECM as cells move across the plate (Fig 1C). Collectively, these data suggest the recombinant versican is synthesized, secreted, and deposited into the ECM in the versican-transfected fibroblasts.

### Versican alters the phenotype of cultured fibroblasts

**Fig 2:**
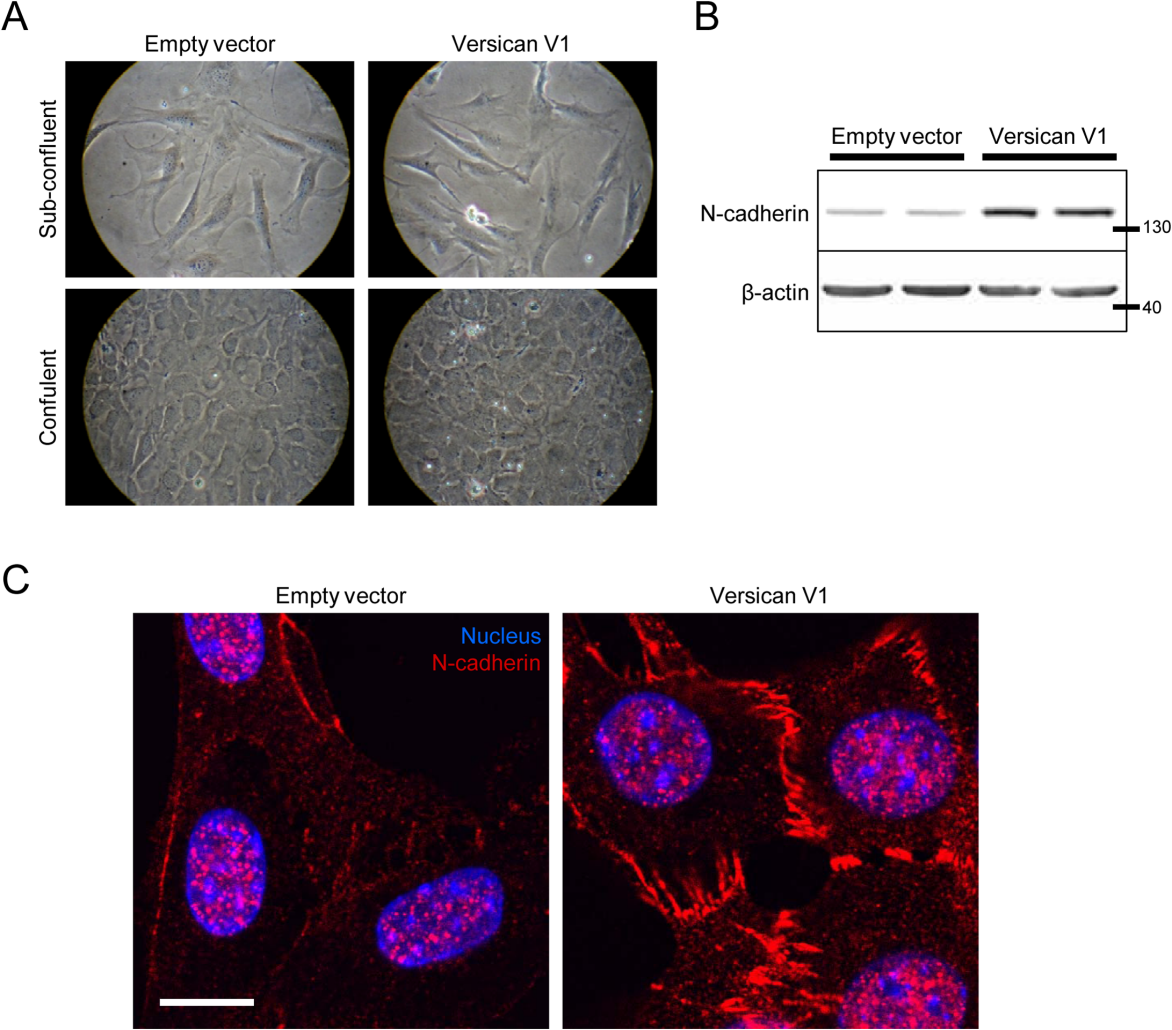
Versican increases N-cadherin expression. (A) Light microscopy shows no major change in cell morphology in versican-transfected fibroblasts at both sub-confluent and confluent densities. (B) Representative Western blot showing increased expression of N-cadherin in versican-transfected cells. (C) Confocal microscopy confirmed the increased N-cadherin expression in versican-transfected cells. (Scale bar = 12.00 μm).

A recent report has suggested that versican induced the formation of epithelial-like islands in cultured fibroblasts that were characterized by decreased N-cadherin expression. We did not detect a noticeable difference in the morphology of versican-transfected cells when cultured on plastic dishes at either sub-confluent or confluent cell densities (Fig 2A). Interestingly, we actually observed N-cadherin expression to be increased in the versican expressing fibroblasts, as shown by Western blot (Fig 2B, 4.11 ± 0.71 fold increase, p<0.05) and immunofluorescent staining (Fig 2C).

**Fig 3:**
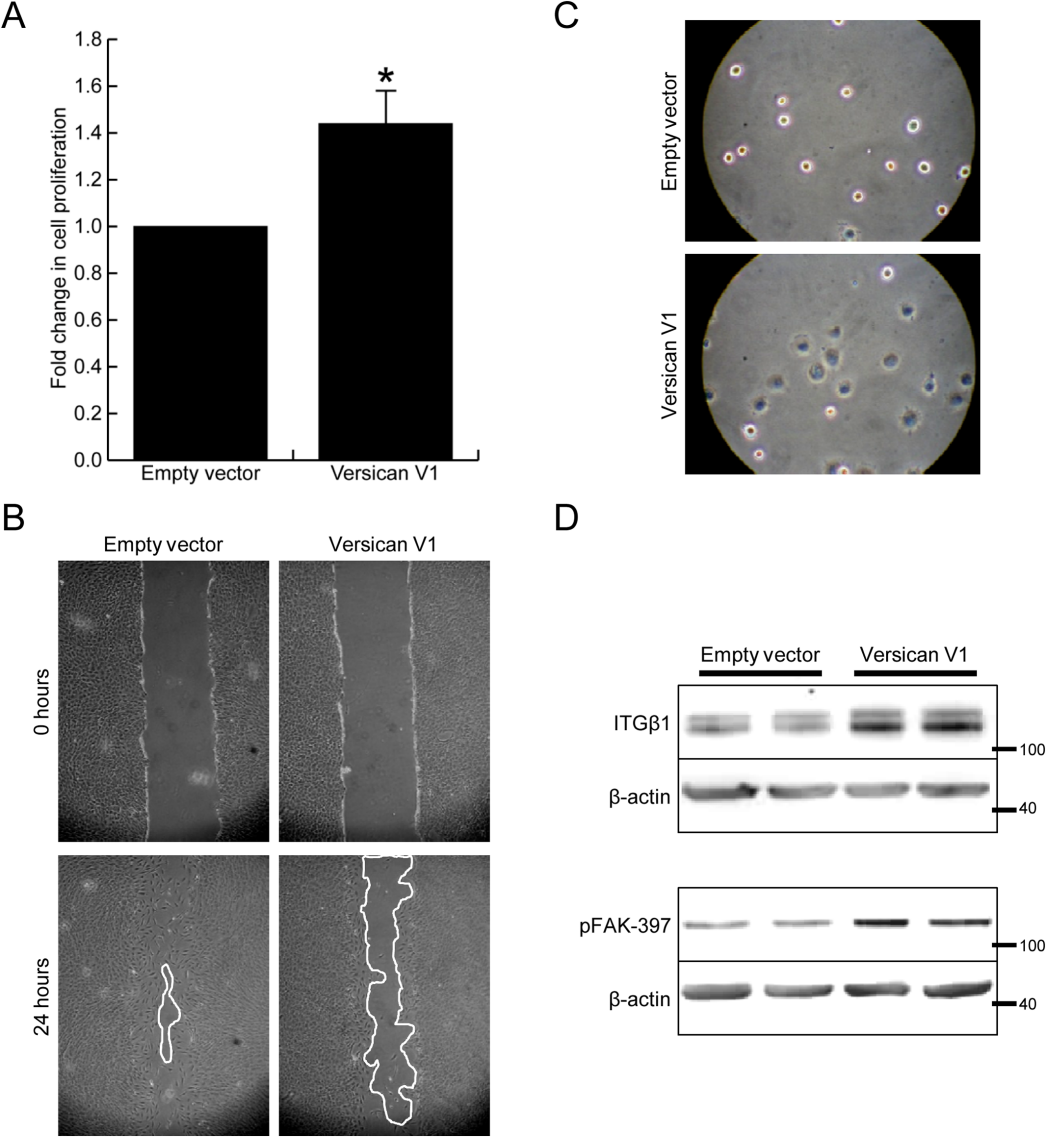
Versican alters the function of fibroblasts. (A) Versican increased cell proliferation 1.44 ± 0.07 fold over control cells, p<0.05. (B) Versican expressing fibroblasts showed decreased cell migration, as measure by in vitro scrape wound assay. (C) Cell adhesion was found to be increased in versican-transfected fibroblasts, measured at 30 minutes after seeding. (D) Representative Western blot showing increased integrin β1 expression, as well as phosphorylation of FAK-397, in versican-transfected fibroblasts. (* denotes p<0.05).

Although no major change in morphology was observed, the versican-expressing fibroblasts were found to function much differently than control cells. The versican fibroblasts proliferated significantly faster than control cells (Fig 3A, relative rate = 1.44 ± 0.07 of control cells, p<0.05). In contrast, the versican fibroblasts migrated slower than control cells (Fig 3B, 65 ±12 vs 96 ±3 percent wound closure, p<0.05). These cells also showed increased adhesion to culture dishes, as measured by cell spreading 30 minutes after seeding (Fig 3C, 3.43 ± 0.63 fold increase in cell surface area, p<0.05). Concomitant with increased cell adhesion, the versican fibroblasts displayed increased expression of integrin β1 as well as phosphorylation of focal adhesion kinase (FAK) (Fig 3D, 2.47 ± 0.41 and 2.01 ± 0.44 fold increase respectively, p<0.05).

### Versican induces a myofibroblast-like phenotype in cultured fibroblasts

**Fig 4:**
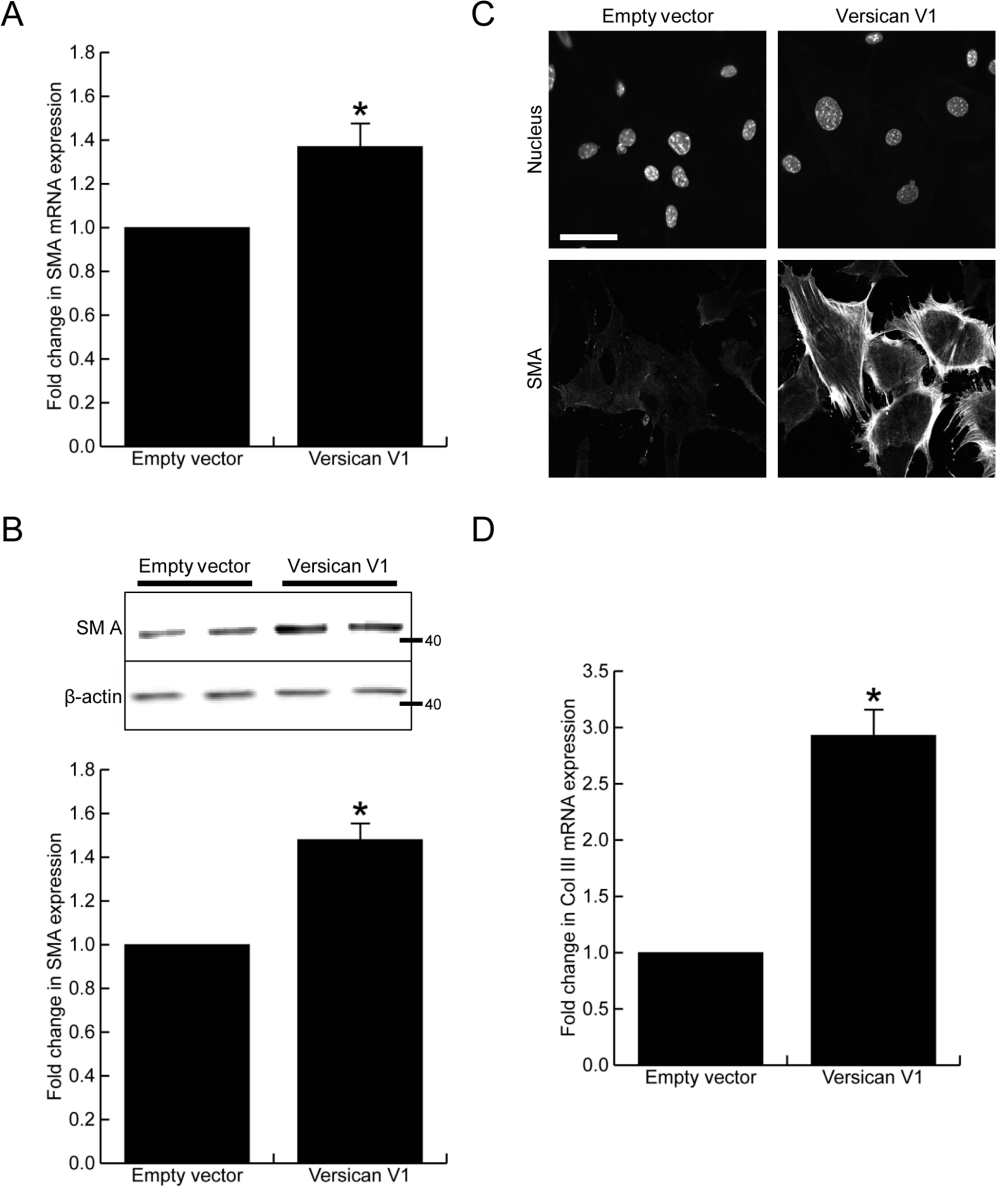
Versican increases smooth muscle α-actin expression. (A) Versican induced a 1.38 ± 0.15 fold increase in smooth muscle α-actin mRNA expression, p<0.05. (B) Versican induced a 1.47 ± 0.10 fold increase in smooth muscle α-actin protein expression, p<0.05. (C) Immunofluorescent staining confirmed smooth muscle α-actin expression was increased in the versican-transfected cells. (D) Versican induced a 2.93 ± 0.22 fold increase in collagen III mRNA expression, p<0.05. (Scale bar = 47.00 μm, * denotes p<0.05)

Many reports have shown versican expression to be in close association with myofibroblasts in injured tissues, therefore we next investigated whether versican altered smooth muscle α-actin, a commonly used marker of myofibroblast differentiation. Versican significantly increased smooth muscle α-actin mRNA (Fig 4A, 1.38 ± 0.15 fold increase, p<0.05) and protein expression, the latter shown by Western blot (Fig 4B, 1.47 ± 0.10 fold increase, p<0.05) and immunofluorescent staining (Fig 4C). In addition to the increased smooth muscle α-actin expression, the versican fibroblasts had a 2.93 ± 0.22 fold increase in collagen III mRNA levels when compared to control cells (Fig 4D, p<0.05).

**Fig 5:**
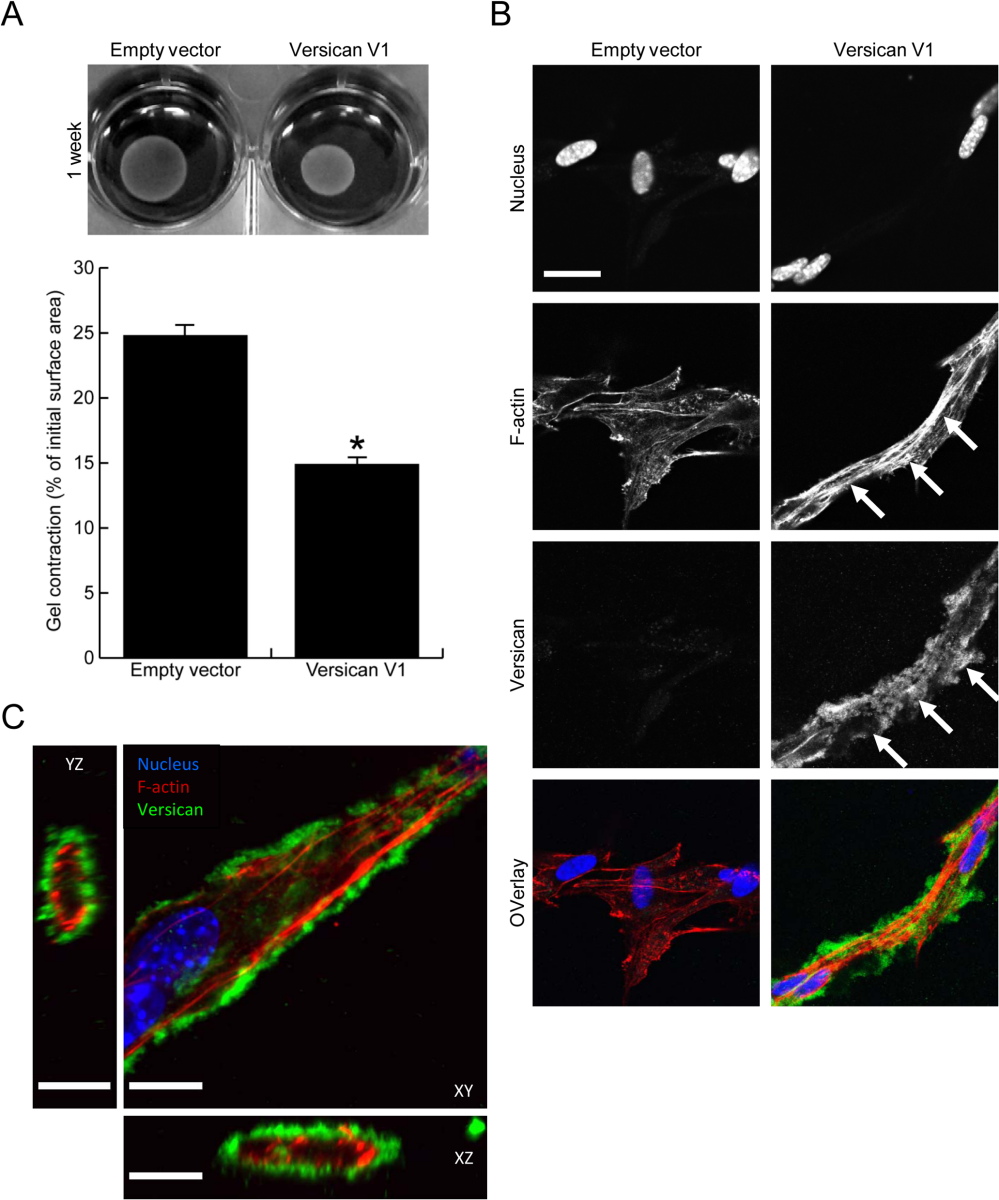
Versican increases fibroblast-mediated contraction of a collagen lattice. (A) Representative images of contracted collagen gels are shown along with the quantified surface areas of contracted gels. Versican significantly increased the fibroblast-mediated contraction of collagen gels (14.9 ± 0.7% vs 24.8 ± 1.8% of initial gel area, p<0.05). (B) Confocal imaging demonstrated the versican-transfected fibroblasts to be elongated, interconnected, and to have increased stress fibre formation in collagen gels (arrows, red in overlay). Versican was found to localize to the pericellular matrix surrounding elongated cells (arrowheads, green in overlay). (C) A Z-stack image reveals versican (green) forms a pericellular coat around cell protrusions in versican-transfected fibroblasts, suggesting it may be well-situated to influence biological events at the cell membrane. (Scale bars = 23.00 μm in B, 12.00 μm in C, * denotes p<0.05)

To investigate whether the increased contractile protein expression seen in versican expressing fibroblasts translated functionally into increased contractile properties, we employed a collagen gel contraction assay. Versican-transfected fibroblasts (or empty vector control cells) were seeded into 0.5 mg/mL type I collagen gels, and contraction was monitored over the period of 7 days. Representative images and the quantified surface area of contracted gels are shown in Fig 5A. Versican expression significantly increased fibroblast-mediated contraction of the type I collagen gels as compared to control cells (14.9 ± 0.7% vs 24.8 ± 1.8% of initial gel area, p<0.05). Immunofluorescent staining was performed on versican and control fibroblasts cultured in the 3 dimensional collagen gels and in this setting a clear change in cell phenotype was observed in the versican expressing cells. These cells exhibited an elongated morphology, were interconnected and had clearly visible f-actin stress fibres, while the control cells did not display prominent cell protrusions or stress fibres (Fig 5B). Versican was found to form a pericellular coat around the cell membrane in the versican-transfected cells that was clearly visible in Z-stack confocal images as a ring around cell protrusions (Fig 5C), suggesting it may be well localized to influence the bioavailability or activity of growth factors or cytokines present in the pericellular environment.

### Versican mediates increased TGF-β signaling in cultured fibroblasts

**Fig 6:**
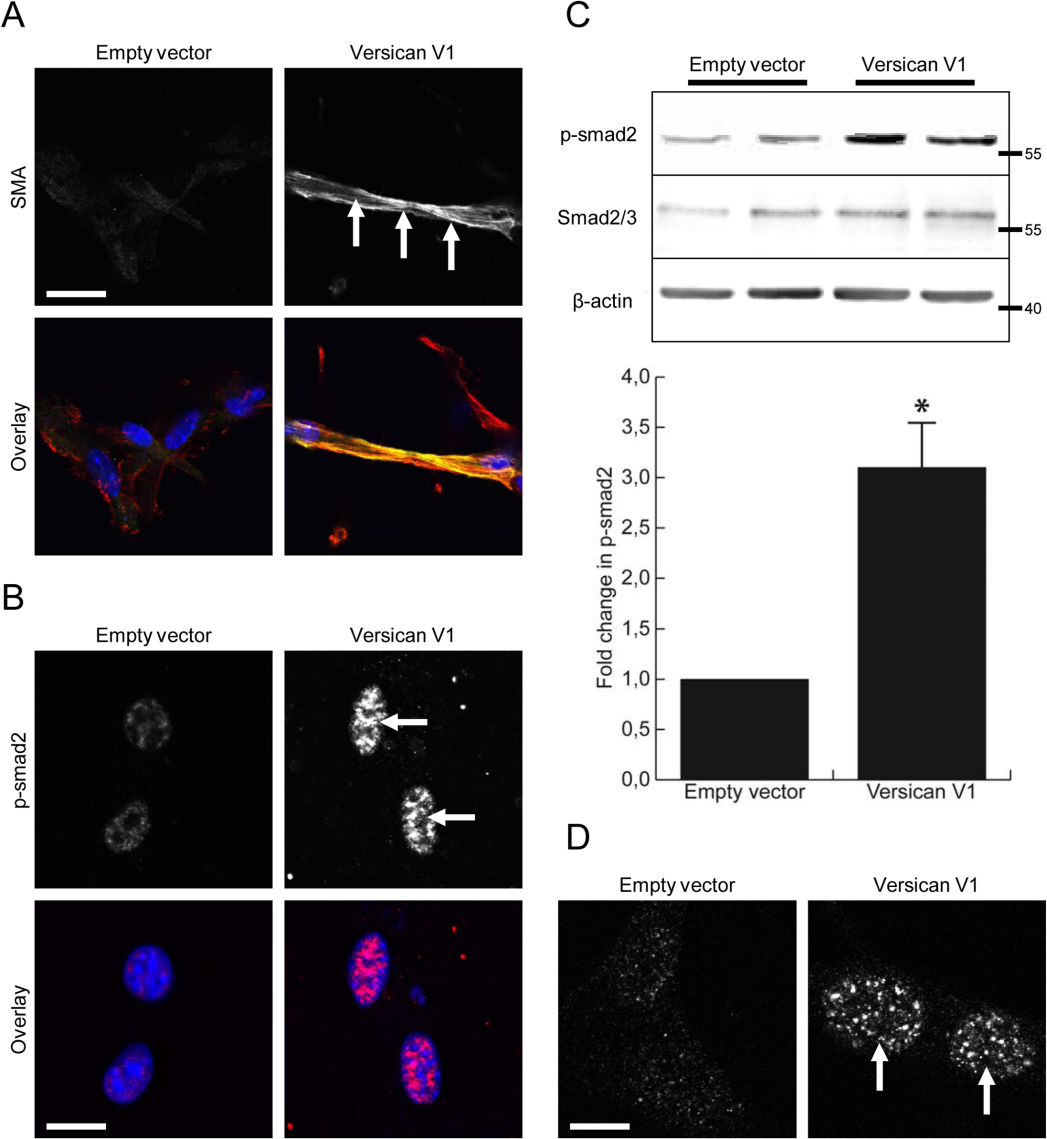
Versican increases TGF-β signaling in cultured fibroblasts. (A) Confocal imaging of contracted gels demonstrated the versican-transfected fibroblasts to have increased expression and incorporation of smooth muscle α-actin into their stress fibres (arrows, yellow in overlay). (B) The versican-transfected fibroblasts also displayed increased staining and nuclear localization of phophorylated SMAD2 in contracted collagen gels (arrows, red in overlay). (C) Representative Western blot shows increased phosphorylation of SMAD2 in cultures of versican-transfected fibroblasts (3.13 ±0.61 fold increase, p<0.05). (D) Confocal microscopy confirmed the increased SMAD2 phosphorylation and nuclear accumulation in cultures of versican-transfected fibroblasts (arrows). (Scale bars = 23.00 μm in A, 12.00 μm in B, D)

A recent report has suggested versican localizes TGF-β and increases its signaling in chondrocytes during joint formation [20]. Therefore, we hypothesized that versican may also alter the TGF-β signaling axis in cultured fibroblasts. Immunofluorescent staining demonstrated increased expression and incorporation of smooth muscle α-actin in versican-transfected fibroblasts that were cultured in type I collagen gels (Fig 6A). These cells also displayed nuclear accumulation of phosphorylated SMAD2, a direct target of TGF-β signaling, when cultured in collagen gels (Fig 6B). To confirm activation of TGF-β signaling in versican expressing fibroblasts, Western blotting was performed on cultured fibroblasts and representative blots are shown in Fig 6C. The versican expressing fibroblasts displayed increased SMAD2 phosphorylation as compared to control cells (3.13 ±0.61 fold increase, p<0.05). The increased nuclear accumulation of phosphorylated SMAD2 was again observed in 2D cultures of versican-transfected fibroblasts, confirming activation of this signaling pathway (Fig 6D).

## Discussion

In this study, we cloned the full length V1 isoform of human versican and expressed the recombinant protein in mouse fibroblasts. Using this system, we found the versican-transfected fibroblasts to have an altered cell phenotype when compared with the empty vector control cells. Functionally, the versican expressing fibroblasts proliferated faster and displayed increased cell adhesion, but migrated slower than control cells. These changes in cell function were associated with increased N-cadherin and integrin β1 expression, along with increased FAK phosphorylation. In addition, the versican expressing fibroblasts displayed increased expression of smooth muscle α-actin, a marker of myofibroblast differentiation [21]. Consistent with these changes, the versican fibroblasts had increased synthetic activity, as measure by collagen III mRNA expression, as well as an increased capacity to contract a collagen lattice. These phenotypic changes appear to be mediated, at least in part, by versican increasing TGF-β signaling through SMAD2. Collectively, these data suggest a role for versican in promoting myofibroblast differentiation in cultured fibroblasts.

Growing evidence suggests versican is associated with normal and aberrant injury and repair events, but the function of this proteoglycan remains poorly understood. Versican has consistently been shown to be expressed in injured and healing tissues, including after skin injury by incision [22] or burn [23], following myocardial infarction [7], in stented arteries [2,3] and in cancers [10,24,25], among others. Other groups have reported the accumulation of a versican- and hyaluronan-rich ECM around actively proliferating and migrating mesenchymal cells [26,27], and a knockdown of versican in smooth muscle cells inhibited these processes [10,28]. In these settings, versican expression appears to be regulated by growth factor activation of receptor tyrosine kinase signaling. Versican is dramatically upregulated by PDGF [29,30] and TGF-β [8,12,31,32], and our lab has previously shown a role for Wnt signaling through β-catenin in regulating versican expression [33]. Our data add to this story by suggesting versican promotes the formation of a contractile, myofibroblast-like phenotype in cultured fibroblasts with increased expression of smooth muscle α-actin. In addition, versican was shown to alter the proliferation, migration and adhesion of cultured fibroblasts. As these cell-mediated processes are all critically involved in a wound healing response, these data suggest versican may influence the outcomes of repair. When taken together, these studies suggest locally released growth factors and cytokines that are released at sites of injury promote the formation of a versican-rich ECM that regulates the phenotype of the cells embedded within it.

In addition to the increased smooth muscle α-actin expression, our data show that versican increases the levels of integrin β1 and phosphorylated FAK in cultured fibroblasts. This finding is consistent with previous work in which a ‘mini’ versican construct or expression of its c-terminal globular domain increased integrin β1 levels in an astrocytoma cell line [34,35]. In an elegant series of papers in the setting of fibrotic lung disorders, Bensaduon et al demonstrated that collagen synthesis takes place in a versican-rich provisional matrix during early repair events, and suggested versican initiates the process of matrix remodeling following lung injury [36,37]. Our data are in support of this idea, as we observed increased collagen III mRNA expression in the versican-transfected fibroblasts as well as increased contractile properties in these cells. The upregulated expression of integrin signaling seen in the versican fibroblasts may provide a mechanism to facilitate cellular interaction with the early collagen matrix. How versican increases integrin expression is not clear from the present study, although the localization of versican to the pericellular space in three-dimensional gels suggests it is well situated to modify a number of biological processes that are occurring at the cell membrane and may be involved. For example, versican has been shown to stimulate MAPK signaling by directly activating the EGFR through two EGF-like repeats in its C-terminal domain [38,39]. Versican has also been known to bind growth factors and may regulate their activity [40]. In support of this, it has been shown that versican is necessary to localize TGF-β in the pericellular matrix and thereby regulates its signaling during chondrogenesis [20]. Our data are consistent with this finding, as we observed increased phosphorylation and nuclear accumulation of SMAD2, a downstream signaling target of TGF-β, in the versican expressing fibroblasts. Recently, a report was published in which fibroblasts isolated from ADAMTS5 knockout mice (versican protease) were found to develop myofibroblast-like properties [41]. In their study, Hattori et al showed that accumulation of versican in the pericellular matrix, either by knocking out ADAMTS5 or overexpressing versican, led to increased smooth muscle α-actin expression, contractility of collagen gels and higher TGF-β signaling, data which is in agreement with our findings. Further, they demonstrate in their study that reducing versican ameliorates the myofibroblast properties of fibroblasts. As TGF-β is the most potent known inducer of myofibroblast differentiation [42], it is likely that activation of its signaling is responsible for the myofibroblast-like phenotype observed in fibroblasts cultured in a versican-rich matrix.

Interestingly, a previous report in which versican was expressed in NIH3T3 fibroblasts showed that it promoted an epithelial-like phenotype in these cells which was characterized by decreased N-cadherin expression [43]. In the study, versican expressing fibroblasts were found to aggregate and form ‘epithelial islands’ that were distinctly observable by light microscopy. In our study, we did not observe the formation of epithelial-like aggregates in our model of versican expression, and actually observed N-cadherin expression to be increased. Versican is a complex molecule which forms numerous interactions with other proteins in the matrix and it is difficult to determine the cause of these discrepancies. It should be noted that chicken versican was used in the previous study whereas we used human versican in this work. It has been shown that sequence variation surrounding the GAG attachment sites may affect the priming and modification of the attached GAG chains, and this could be one possibility for the observed differences. It is beyond the scope of the current article to analyze and compare the fine structure of the versican GAG chains, but clearly more work is necessary to better understand the effects of versican on cell behavior.

In summary, we have shown that versican promotes the formation of a myofibroblast-like cell phenotype in cultured fibroblasts. These results lend support to the idea that versican is an important component of the provisional wound healing matrix that is expressed following injury. However, if versican also promotes myofibroblast differentiation and increased contractile properties *in vivo*, the relative amount of versican expressed at sites of chronic inflammation could promote excessive tissue remodeling and fibrosis.
